# Rapid response of Nelson’s syndrome to pasireotide in radiotherapy-naive patient

**DOI:** 10.1186/s40842-020-00110-7

**Published:** 2020-11-07

**Authors:** Xin He, Joanna L. Spencer-Segal

**Affiliations:** 1 Department of Internal Medicine, Division of Metabolism, Endocrinology & Diabetes, University of Michigan, 1500 East Medical Center Drive, Ann Arbor, MI 48109 USA; 2 Michigan Neuroscience Institute, University of Michigan, 205 Zina Pitcher Pl, Ann Arbor, MI 48109 USA

**Keywords:** Nelson’s syndrome, Cushing’s disease, Pasireotide, Somatostatin analog

## Abstract

**Background:**

Nelson’s syndrome is a well-described complication following bilateral adrenalectomy for management of Cushing’s disease. There is no consensus on optimal management of Nelson’s syndrome, characterized by the triad of pituitary corticotroph adenoma growth, elevated serum adrenocorticotropic hormone, and skin hyperpigmentation. Medical therapy with a variety of drug classes have been studied. One potentially promising drug already approved for Cushing’s disease is pasireotide, a somatostatin analog with affinity for multiple somatostatin receptors, including subtype 5, the most highly expressed receptor on corticotroph tumors.

**Case presentation:**

A 24-year-old female was diagnosed with Cushing’s disease with initial ACTH levels around 700–800 pg/mL. She underwent transsphenoidal surgery without remission, followed by bilateral adrenalectomy. Over the subsequent 3 years, the patient developed skin hyperpigmentation, recurrent elevations of ACTH, and tumor recurrence requiring two additional transsphenoidal surgeries. After her third transsphenoidal resection, ACTH normalized, no residual tumor was seen on radiology, and the patient’s skin hyperpigmentation improved. She then had an uncomplicated full-term pregnancy, during which ACTH levels remained within normal limits. One month after delivery, ACTH levels began rising to a peak at 5,935 pg/mL. Imaging revealed two new bilateral pituitary adenomas, measuring 14 mm on the left, and 7 mm on the right. She was then started on pasireotide. After two months of therapy, ACTH decreased to 609 pg/mL, and repeat pituitary MRI showed interval decrease in size of both pituitary adenomas to 13 mm on the left and 6 mm on the right.

**Conclusion:**

We report the protracted course of a young female with several recurrences of Nelson’s syndrome following bilateral adrenalectomy and multiple transsphenoidal surgeries, who ultimately responded to pasireotide. Unique features of her case not described previously are the response to pasireotide in a radiotherapy-naive patient, as well as the rapid radiologic response to therapy. Her history illustrates the unresolved challenges of Nelson’s syndrome and the continued need for additional studies to identify optimal management.

## Background

Nelson’s syndrome is a well-described complication following bilateral adrenalectomy for management of Cushing’s disease. While the preferred first-line management of Cushing’s disease is surgical resection of the underlying corticotroph tumor, bilateral adrenalectomy can be an effective alternative when the underlying lesion is unidentifiable, unresectable, or refractory to surgery. In 88–100% of cases, adrenalectomy results in successful remission of Cushing’s disease and control of hypercortisolism [1, 2]. However, studies of surgical cohorts indicate that 15% to 47% of patients experience growth of pituitary corticotroph adenomas, elevated serum adrenocorticotropic hormone (ACTH), and skin hyperpigmentation [1, 3–7]; these three features are considered the triad of Nelson’s syndrome.

The clinical presentation and time course of Nelson’s syndrome can be highly variable, ranging from mild hyperpigmentation occurring 6 months after adrenalectomy, to severe neurologic symptoms related to tumor mass effect developing after two decades [1, 5, 3, 6–8]. There is currently no consensus on the management of Nelson’s syndrome. Management options include observation, surgical resection, pituitary radiotherapy, and medical treatment.

Medical therapy with a variety of drug classes have been studied with inconsistent effectiveness [9–11]. One potentially promising drug is pasireotide, a somatostatin analog with affinity for somatostatin-receptor subtypes 1, 2, 3, and 5 [12]. Pasireotide was found to inhibit ACTH production in vitro and in vivo [13], ultimately leading to its FDA-approval for treatment of Cushing’s disease in 2012 [14]. Here, we present a case of recurrent Nelson’s syndrome with rapid and impressive response to pasireotide in a radiotherapy-naïve patient.

## Case presentation

**Fig. 1:**
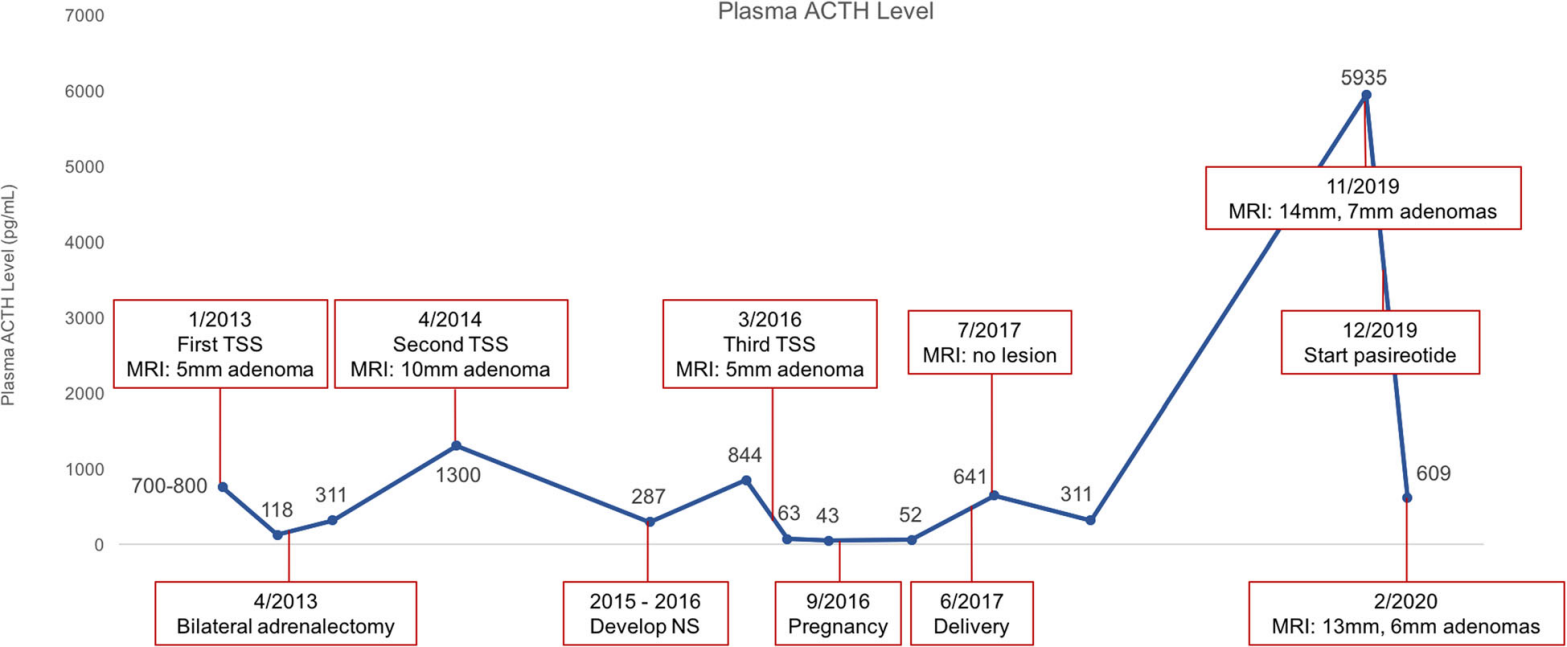
Plasma ACTH levels plotted over time. Major clinical events and imaging findings are indicated. TSS, transsphenoidal surgery; NS, Nelson’s syndrome

A 24-year-old female presented to an outside medical center and was diagnosed with ACTH-dependent Cushing’s disease due to a 5 mm pituitary microadenoma located in the midline inferior portion of the pituitary. At the initial diagnosis, ACTH levels were around 700–800 pg/mL (Fig. 1). She underwent transsphenoidal surgery without remission: postoperative cortisol was documented as 18 mcg/dL with ACTH 118 pg/mL, prompting eventual bilateral adrenalectomy three months later.

Over the subsequent year, the patient’s ACTH level gradually increased up to a peak of 1300 pg/mL. Repeat pituitary imaging revealed a 10 mm pituitary adenoma in the same location as her initial microadenoma, prompting a second transsphenoidal surgery. Pituitary MRI completed six months after her second transsphenoidal surgery showed postsurgical changes in the posterior superior nasal septum without evidence of a sellar mass. However, postoperative ACTH levels ranged 200–300 pg/mL.

**Fig. 2:**
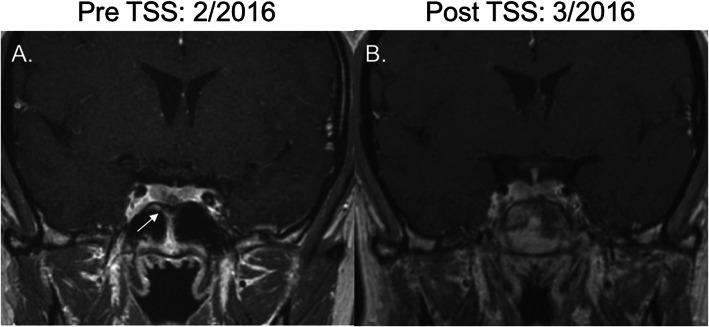
Coronal T1-weighted MRI scans. **a**. February 2016: Inferior pituitary 5 mm lesion indicative of recurrence of Nelson’s syndrome. Imaging completed prior to third transsphenoidal surgery. **b**. March 2016: Resolution of pituitary lesion after third transsphenoidal surgery

Over the subsequent 2 years following her second transsphenoidal surgery, the patient noted progressive darkening of her skin and recurrent episodes of sinusitis. Repeat pituitary MRI revealed a 5 mm microadenoma, again in the midline inferior region of the pituitary gland (Fig. 2a). Labs included ACTH 855 pg/mL with cortisol 18 mcg/dL. The patient was referred to our clinic at this point. On our evaluation, we noted hyperpigmentation of her lips, axillae, and adrenalectomy scars. After discussion of the treatment options, the patient elected to undergo a third transsphenoidal surgery, and this was completed without complications (Fig. 2b). The resected tumor was found to be an adenoma staining positive for synaptophysin and ACTH with an elevated Ki67 proliferation index of 8%. Postoperatively, ACTH normalized to a nadir of 43 pg/mL. The patient’s skin hyperpigmentation also improved. She had no evidence of developing hypopituitarism after surgery: TSH, free T4, and sodium levels remained normal, and menses occurred regularly.

Six months after her third transsphenoidal surgery, the patient conceived spontaneously. ACTH during her second trimester was in the upper end of normal range, 52 pg/mL. The patient vaginally delivered a healthy boy at 39 weeks gestation without complications. ACTH checked one month after delivery was elevated at 641 pg/mL. The patient again developed skin hyperpigmentation. Pituitary MRI did not show any lesions. Radiation therapy and medical therapy were discussed with the patient, but treatment was postponed as she was breastfeeding. At follow-up nine months postpartum, the patient had stopped breastfeeding. ACTH was 311 pg/mL and surveillance was continued.

**Fig. 3:**
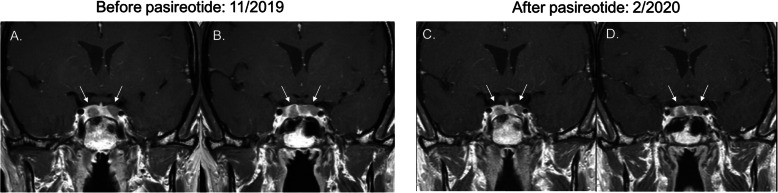
Coronal T1-weighted MRI scans. **a** and **b**. November 2019: Bilateral pituitary lesions measuring 14 mm and 7 mm indicative of recurrence of Nelson’s syndrome. Pasireotide was initiated one month after this imaging was completed. **c** and **d**. February 2020: Bilateral pituitary lesions measuring 13 mm and 6 mm after 2 months of pasireotide treatment

Over 3 years after her third transsphenoidal surgery, the patient returned for follow-up with progressive skin darkening. ACTH rose to 5,935 pg/mL. Pituitary MRI revealed interval enlargement of the pituitary gland, as well as development of two new nonenhancing adenomas, 14 mm on the left extending to midline, 7 mm on the right (Fig. 3a and b). Treatment options of transsphenoidal surgery, radiation therapy, and pasireotide were discussed. The patient elected to start pasireotide. She had already undergone placement of an intrauterine device for contraception.

After 1 month of taking short-acting pasireotide 0.3 mg twice daily, the patient reported side effects of mild diarrhea and nausea. She also developed frequent palpitations, for which a Holter monitor was ordered and was unrevealing. She noted improvement in her skin hyperpigmentation, notably that she was able to better visualize her pre-existing tattoos. ACTH level two months after starting pasireotide decreased to 609 pg/mL (Fig. 2). Repeat pituitary MRI also showed slight interval decrease in size of both pituitary adenomas to 13 mm on the left extending to midline, 6 mm on the right (Fig. 3c and d). The pasireotide dose was increased to 0.6 mg twice daily. Blood work completed several months after starting pasireotide showed serum glucose levels remaining in normal range with glycated hemoglobin level of 5.2%.

## Discussion and conclusions

More than half a century after Dr. Don Nelson first described the eponymous condition [15], Nelson’s syndrome remains challenging to manage. Current strategies include observation, surgical resection, prophylactic and treatment radiotherapy, and medical management. After bilateral adrenalectomy for treatment of Cushing’s disease, predicting which of the 15–47% of the patients will develop Nelson’s syndrome is difficult [1, 3–7]. Risk factors associated with increased incidence of Nelson’s syndrome are residual pituitary tumor seen on imaging prior to adrenalectomy [6, 16–18] and elevated ACTH 1 year after adrenalectomy [3, 6, 19]. Pregnancy is not associated with accelerated ACTH rise or corticotroph tumor growth [20]. There is inconclusive and/or conflicting data on the association of other risk factors, such as age [3, 21–23], duration of Cushing’s disease prior to adrenalectomy [1, 2, 18, 23], tumor proliferation index [24, 25], elevated urinary free cortisol prior to surgery [1, 6, 18], and inadequate glucocortiocoid replacement postoperatively [1, 26, 27]. Possible contributing risk factors in this patient’s case include her young age, ACTH level of more than 1,300 ng/dL within a year after adrenalectomy, and elevated Ki67 proliferation index; we also cannot exclude the possibility of residual pituitary tumor prior to adrenalectomy, as we do not have imaging from that time. Consistent with existing data in radiotherapy-naïve women [20], the patient’s Nelson’s syndrome was controlled and did not cause complications during pregnancy.

Several studies have evaluated the efficacy of prophylactic pituitary radiotherapy after adrenalectomy. Two retrospective reviews found pituitary radiotherapy to be associated with lower incidence of Nelson’s syndrome: one cohort found that 50% of patients who did not receive radiotherapy developed Nelson’s syndrome compared to 0% in the cohort that did [17]; another study found radiotherapy was associated with 25% incidence of Nelson’s syndrome compared to 50% in the absence of radiotherapy [16]. However, several other studies did not find this association [18, 19, 28]. While some centers administer prophylactic radiotherapy in all patients following bilateral adrenalectomy who have residual pituitary tissue [29], this is not universal practice and was not done in this case.

Administration of radiotherapy after onset of Nelson’s syndrome has also been studied, with stereotactic radiosurgery as the most common intervention [9]. Several studies have found that stereotactic radiosurgery is associated with decreasing Nelson’s syndrome tumor volume and/or serum ACTH levels, through response to therapy has been widely variable, with reported long-term remission rates ranging from 14 to 92% [30–35]. Complications of hypopituitarism and partial or permanent cranial nerve damage are seen in 7–40%, 20%, and 5–7% of patients, respectively [31, 35]. To prevent these potential complications in a young woman, radiotherapy was avoided in our patient.

When feasible, surgical resection of the Nelson’s syndrome tumor is commonly recommended. Reported success rates of pituitary surgery varies widely from 10 to 70% [11, 36–38]. Higher rates of remission have been associated with smaller tumors, adjuvant radiotherapy, and absence of tumor invasion into the dura mater [11, 36, 37]. The most common complications are hypopituitarism, cerebrospinal fluid leak, and meningitis occurring in up to 69%, 15%, and 8% of postoperative patients, respectively [38–40]. Patients requiring multiple pituitary surgeries, as was the case in our patient, are not uncommon in the literature [11, 26, 37].

Various classes of medical therapy have been explored but data remain limited. Several case reports found that cabergoline provided long-term control of Nelson’s syndrome, but bromocriptine did not [41–43]. Two case reports have also described the use of temazolomide in aggressive Nelson’s syndrome tumors to successfully achieve significant decreases in tumor size and ACTH level [44, 45]. Rosiglitazone has been studied, as peroxisome proliferator-activated γ receptors are expressed in corticotroph adenomas; however, several small prospective trials showed no ACTH response to rosiglitazone [46–48]. Valproic acid has also been evaluated due to its inhibition of γ-aminobutyric acid, thereby reducing production of corticotropin-releasing hormone by the hypothalamus; however, several small prospective and retrospective studies of valproic acid alone or in conjunction with radiotherapy and surgery have not found consistent decreases in tumor size and ACTH level [17, 49, 50].

Octreotide and pasireotide are somatostatin analogs that have been trialed with encouraging results in Nelson’s syndrome. The first evidence for the use of this drug class was noted when somatostatin infused in a small group of Nelson’s patients decreased their serum ACTH levels by 40% to 71% [51]. Several case series and case reports have found biochemical and symptomatic improvement with octreotide [52–54], with reported decreases in ACTH levels by 54% to 70%. Another case report described more impressive results in a patient with history of Nelson’s syndrome refractory to multiple pituitary surgeries and radiotherapy, for whom pasireotide decreased serum ACTH from 42,710 pg/mL to 4,272 pg/mL, along with decrease in tumor size and improvement in hyperpigmentation; the patient did also develop the known side effect of hyperglycemia [55]. Corticotroph tumors have been found to express somatostatin receptors (SST) 1, 2, 4, and 5, but with the highest expression of SST 5 [56]. Octreotide binds most strongly to SST 2 and with lower affinity to SST 5, whereas pasireotide binds to SST 1, 2, 3, and 5, with high affinity [12]. This differential binding affinity for SST 5 is conjectured to be the reason for improved response of Cushing’s disease [57], and perhaps of Nelson’s syndrome as well, to pasireotide compared to octreotide.

Most recently, a prospective study of pasireotide twice daily injection for four weeks followed by long-acting release formulation for 24 weeks recruited eight patients with Nelson’s syndrome [58]. Six patients overall had partial or complete biochemical response, without conclusive changes in pituitary tumor size or skin pigmentation. Due to side effects including gastrointestinal distress and hyperglycemia, only half (four) of the patients completed the study. Notably, all three patients who had a complete response also had history of receiving radiotherapy prior to study enrollment. The authors suggest that studies of larger cohorts and longer study duration, while balancing the impact of side effects, are needed to better understand the role of pasireotide for management of Nelson’s syndrome.

Unique features of our case not described previously are the response to pasireotide in a radiotherapy-naive patient, as well as the rapid radiologic response to therapy. Due to her young age, radiotherapy had been avoided in management, whereas all existing reports of patients with sustained response to pasireotide received prior radiotherapy. Furthermore, radiologic improvement was observed after just two months of pasireotide therapy; radiologic improvement was noted on the nine-month follow-up imaging in the prior case report [55], but not seen in the prospective trial [58]. Thus, this is the first report of a radiotherapy-naïve patient with Nelson’s syndrome who has responded to pasireotide.

In summary, we report the protracted course of a young female with Nelson’s syndrome with recurrence after multiple transsphenoidal surgeries, who ultimately responded to pasireotide. Like the participants in the prospective study, our patient similarly has experienced a number side effects, and it remains to be seen whether she will tolerate pasireotide therapy long term. Her history illustrates the unresolved challenges of Nelson’s syndrome and the continued need for additional studies to identify optimal management.

## Data Availability

Not applicable.

## Abbreviations

ACTH: Adrenocorticotropic hormone
NS: Nelson’s syndrome
SST: Somatostatin receptor
TSS: Transsphenoidal surgery
TSH: Thyroid stimulating hormone
